# Rowing through recovery: Psychophysical outcomes of a combined 12-week rowing and exercise program in breast cancer survivors

**DOI:** 10.1007/s00520-026-10361-2

**Published:** 2026-01-23

**Authors:** María Del Rosario Asensio-Garcia, Sergio Hernández-Sanchez, Emilio Jose Poveda-Pagan, Rauf Nouni-Garcia, Jose Vicente Segura-Heras, María Isabel Tomas-Rodriguez

**Affiliations:** 1 Department of Pathology and Surgery, Center for Translational Research in Physiotherapy, Miguel Hernández University, Alicante, San Juan de Alicante Spain; 2 Rehabilitation Service, University Hospital of San Juan de Alicante, Alicante, San Juan de Alicante Spain; 3 Department of Clinical Medicine, Miguel Hernández University, Alicante, San Juan de Alicante Spain; 4 Instituto Centro de Investigación Operativa, Miguel Hernández University, Alicante, San Juan de Alicante Spain; 5 Department of Pathology and Surgery, Physiotherapy Area. Center for Translational Research in Physiotherapy, Miguel Hernández University. Campus Sant Joan. Crta Valencia, S/N. 03550. San Juan de Alicante, Alicante, Spain

**Keywords:** Breast cancer survivors, Rowing, Physical activity, Fatigue, Quality of life, Group exercise

## Abstract

**Purpose:**

This study aimed to evaluate the effects of a 12-week multicomponent intervention combining fixed-seat rowing (*falucho*) with targeted strength and flexibility exercises on physical and psychosocial outcomes in women with and without a history of breast cancer.

**Methods:**

A quasi-experimental, parallel-group design was employed. Nineteen breast cancer survivors and nineteen healthy women participated in a structured program consisting of twice-weekly open-water rowing sessions and gym-based conditioning exercises. Functional, psychological, and quality of life variables were assessed at baseline and post-intervention using validated tools (Hand Grip Strength, 30-Second**s** Sit to Stand Test, QuickDASH, Piper Fatigue Scale, and WHOQOL-BREF). A multivariate repeated measures model and bootstrapping methods were used for statistical analysis.

**Results:**

Significant improvements over time were observed in all functional variables across both groups. Notably, breast cancer survivors showed greater reductions in upper limb disability (QuickDASH, p = 0.011) and cancer-related fatigue (Piper score, p < 0.001). Positive effects on quality of life were also found, particularly in the physical dimension. Group-based rowing in an outdoor maritime setting may have contributed to improved emotional well-being and social engagement, although psychological, social, and environmental domains showed only time-related effects. Attendance exceeded 80% of planned sessions, with no adverse events reported.

**Conclusion:**

This intervention appears to be a feasible and beneficial strategy to enhance physical function, reduce fatigue, and support psychosocial recovery in breast cancer survivors. The findings suggest that traditional rowing, especially when combined with complementary exercises, may be a valuable addition to oncology rehabilitation programs.

**Supplementary Information:**

The online version contains supplementary material available at 10.1007/s00520-026-10361-2.

## Introduction

Breast cancer (BC) represents one of the most significant challenges in women's health worldwide [1]. It is the most common neoplasm among women, with an estimated global incidence of 2.3 million new cases in 2022 [2]. This condition not only threatens survival but also profoundly impacts the quality of life of those affected [3]. The physical and psychological sequelae resulting from treatments—such as surgery, radiotherapy, and chemotherapy—may include loss of muscle strength, reduced flexibility, chronic fatigue, and limitations in overall functionality [4, 5]. This scenario underscores the importance of implementing comprehensive therapeutic interventions that support both physical and psychological recovery in this population [6].

Current scientific evidence strongly supports physical exercise as an effective strategy to address complications in female breast cancer survivors and to improve their quality of life [7]. However, despite the demonstrated benefits, it is estimated that two out of three individuals who have had cancer do not meet the minimum physical activity recommendations outlined in current guidelines [8]. These guidelines recommend engaging in at least 150 min of moderate-intensity physical activity per week or 75 min of vigorous-intensity exercise, supplemented with resistance training at least twice weekly [9].

This low adherence highlights the urgent need to implement strategies that promote exercise compliance, such as engaging in group-based exercise, a strategy that has proven effective not only among breast cancer survivors but also among women without a history of the disease [10].

Training programs that combine aerobic and resistance exercises significantly improve physical function in female breast cancer survivors [11]. These programs have demonstrated benefits such as increased aerobic capacity [7], improved maximal oxygen consumption—associated with better cardiovascular health and overall functionality [12] and enhanced muscle strength, which facilitates the performance of daily activities and helps prevent sarcopenia [13].

In this context, rowing is a sport modality that combines aerobic activity and muscular strength, involving alternating and cyclical movements of the limbs, as well as trunk flexion and extension [14]. Recent studies have shown that it is an effective strategy for inducing improvements in joint range of motion, muscle strength, and quality of life in individuals who practice it [14–16]. As such, it has been proposed as a discipline with great potential for addressing the sequelae associated with breast cancer and its treatments.

Among the various rowing modalities, Dragon Boat is the most commonly studied in research addressing its effects on this population of women [17]. It has been shown to be a safe strategy that may help improve quality of life and upper limb function after breast cancer surgery, prevent the development of lymphedema, and enhance cardiac function in individuals who have undergone chemotherapy during cancer treatment [18].

In other types of rowing boats, such as the falucho—a fixed-seat boat characteristic of the Mediterranean Sea—there are some biomechanical differences compared to Dragon Boat, particularly in terms of paddling technique. In the case of the falucho, movements are of smaller amplitude and do not involve overhead gestures, which reduces biomechanical strain and allows for a more harmonious and sustainable cadence [19]. The practice of this rowing modality has also been shown to produce positive effects on physical health and quality of life in breast cancer survivors [14, 20].

Evidence regarding the combined impact of *falucho* rowing programs along with specific strength and flexibility exercises is still emerging, particularly in women with a history of breast cancer [14, 21]. This comprehensive approach may offer significant benefits, not only in the physical domain of health, but also in psychosocial aspects that are essential for holistic recovery.

Therefore, the aim of the present study was to evaluate the effects of a multifaceted intervention program that combines *falucho* rowing with targeted strength and flexibility exercises, analyzing its impact on the physical and psychosocial status of breast cancer survivors in comparison with women with no history of the disease.

## Material & methods

### Study population

The study population consisted of two groups of women: the breast cancer group (52.8 ± 5.9 years) and the healthy control group (60.3 ± 6.6 years). Both groups participated in fixed-seat rowing sessions twice per week, with each session lasting 60 min. Additionally, they followed a 12-week physical exercise program designed to improve flexibility and muscle strength, as detailed in the protocol provided in Appendix 1.

Eligibility criteria included women who had overcome breast cancer and had received neoadjuvant and/or adjuvant therapy as part of their treatment, as well as women with no personal history of breast cancer who engaged in recreational rowing as a form of health-promoting and preventive physical activity.

### Study design

This was a quasi-experimental study with a parallel group comparison design and a follow-up period of 12 weeks.

In March 2024, all women who met the eligibility criteria and were participating in recreational fixed-seat rowing (falucho) at the Real Club de Regatas de Alicante (Alicante, southeastern Spain) were contacted. These participants were already engaged in two one-hour sessions per week (see Appendix 1). The study was explained to all potential participants, and they were invited to take part beginning on April 1, 2024. Detailed information was provided both orally, through an informational session, and in writing.

To participate in rowing activities at the Real Club de Regatas de Alicante, it is mandatory to submit an official medical certificate confirming, through medical testing and electrocardiogram, that the individual does not have any contraindications to this type of physical activity. Additionally, accident insurance and a monthly club membership fee are required.

The study was conducted in accordance with the Declaration of Helsinki and approved by the Ethics Committee of the Sant Joan d'Alacant University Hospital on January 29, 2019 (approval code: 19/302). Participants who agreed to take part signed an informed consent form and completed a set of patient-reported outcome measures following the initial information session (see "Variables and Measurements") and again at the end of the 12-week exercise program.

The following baseline descriptive variables were also collected from all participants: occupation (whether they were currently employed), age, height, weight, oxygen saturation, resting heart rate, and number of children. For participants with a history of breast cancer, additional clinical information was gathered, including type of treatment received (surgery, chemotherapy, and radiotherapy), whether a mastectomy had been performed (right or left), and the presence of lymphedema in the upper limb.

### Outcome measures

The primary variables of the study were obtained from both self-administered questionnaires and several physical function tests. Data were collected at baseline and after the 12-week intervention by a member of the research team. The variables assessed and the instruments used for their measurement are detailed below:

Hand Grip Strength (HGS)

The assessment of hand grip strength is widely used to evaluate muscular function and physical capacity in various clinical populations, including oncology patients [22]. It is a simple, safe, and reliable procedure that provides an objective measure of muscular strength across all age groups [23]. Measurements were performed using a JAMAR® Hydraulic Hand Dynamometer (Model J00105, Lafayette Instrument Company, United States of America) [24]. Participants performed the test while seated, with the dominant arm flexed at a 90° angle and the elbow held close to the body, following the recommendations of the American Society of Hand Therapists [25]. Three consecutive measurements were taken, with a 30-s interval between each trial. The final result was expressed as the average of the three measurements, in kilograms (kg).

30-Seconds Sit to Stand Test (STS_30).

This test evaluates the functional capacity of the lower limbs by measuring how many times a participant can rise from a seated to a standing position in 30 s, using an armless chair (43 cm seat height) [26]. While seated, with feet flat on the floor and arms crossed over the chest, the participant repeatedly stands up and sits down for 30 s. The total number of completed repetitions is recorded. The test has been shown to be highly reproducible and safe, with good correlation to other functional measures such as the six-minute walk test [27].

The Shortened Disabilities of the Arm, Shoulder and Hand Questionnaire (QuickDASH).

This is an abbreviated version of the Disabilities of the Arm, Shoulder and Hand (DASH) questionnaire, designed to assess upper extremity disability in individuals with musculoskeletal conditions. It includes 11 items that evaluate the functional impact of an upper limb injury on daily activities, household/garden work, shopping, recreation, personal care, eating, sleeping, social activities, and work [28]. To calculate the QuickDASH score, at least 10 of the 11 items must be completed. Each item is rated on a 5-point scale, and the final score ranges from 0 (no disability) to 100 (most severe disability). The minimum clinically important difference (MCID) ranges from 9.0 to 11.3 points [29].

Piper Fatigue Scale (PFS).

This multidimensional scale includes 22 items that assess various psychophysical aspects of fatigue. It is one of the most commonly used tools for measuring fatigue in cancer research and includes subdomains for behavioral (6 items), affective (5 items), sensory (5 items), and cognitive/mood (6 items) aspects of fatigue [30]. Each item is scored on an 11-point scale (0–10), with verbal descriptors anchoring the endpoints. Subscale scores are calculated individually and then aggregated to generate an overall fatigue score. Higher scores indicate greater levels of fatigue.

WHOQOL-BREF Quality of Life Scale.

This is a shortened version of the WHOQOL-100, developed to quickly and effectively assess an individual’s subjective perception of quality of life [31]. Widely used and validated in 19 languages, the WHOQOL-BREF provides a quality of life profile across four domains: physical health, psychological health, social relationships, and environment. It consists of 26 items, including two general items on overall quality of life and general health. Each item is rated on a 5-point Likert scale (1 to 5). Domain scores are calculated by averaging the relevant items (range 4–20) and then multiplying the result by 4 to transform it into a scale from 0 to 100, with higher scores indicating a better quality of life.

### Intervention

All women participating in the study followed a falucho open-water rowing training plan, conducted twice per week (a total of 120 min), along with a 12-week physical conditioning program.

The boat used in this study was the falucho, a traditional Mediterranean fixed-seat rowing vessel, propelled by eight rowers and steered by a coxswain. The rowers are seated in pairs, except for the stroke and bow rowers, who occupy individual benches. Each rower handles a single oar, which is attached to the boat with a rope. The rowing technique involves a cyclical, continuous movement performed from a seated position on a fixed bench, engaging the torso, legs, and arms [32].

This technical gesture can be broken down into four sequential phases: catch, drive, finish, and recovery, as illustrated in Video 1

(http://remavida.es/2020/04/28/remavida-y-umh/).

The physical conditioning program was carried out twice per week at the gym facilities of the Real Club de Regatas de Alicante. The intervention included strength, endurance, and mobility exercises targeting the lower limbs [7], trunk [33], and upper limbs [34]. The exercises were performed using a combination of elastic resistance bands of varying intensities, Pilates balls, and bodyweight exercises. A detailed description of the exercise plan is summarized in Appendix 1.

The intervention lasted 12 weeks, with a gradual progression in both volume and intensity. This progression was achieved by progressively increasing the number of repetitions and the resistance level (color) of the elastic bands. a structured and A detailed description of the exercise program are reported in Appendix 2 following the Consensus on Exercise Reporting Template (CERT).

To ensure training continuity, participants who were unable to attend the gym on occasion were offered the option of performing the exercises at home using a specific home-based training guide (Appendix 1). Finally, attendance rates were recorded for each participant, as well as any adverse events related to rowing practice or strength training exercises.

### Statistics

Continuous variables are expressed as means and standard deviations, or median and quartiles if the normality assumption is not met. Categorical variables are expressed as counts and percentages. The chi-square test was used to compare categorical variables and Student’s t-test or Wilcoxon test was used to compare continuous variables. We calculated the effect size using Cohen’s d (continuous variables) or Cramer’s V (categorical variables).

A semi-parametric multivariate repeated measures test has been implemented that assumes neither multivariate normality nor homogeneity of covariance. Bootstrap methods with 5000 simulations are considered for the same, which provide more accurate results in the case of small or moderate sample sizes. An ANOVA statistic for repeated measures with multivariate data (MATS) or univariate data (ATS) has been used.

Values of p < 0.05 were considered statistically significant. All analyses have been carried out with the free software R (R Foundation for Statistical Computing, Viena, Austria).

For sample size calculation, considering a full factorial repeated measures model with one between-subjects factor (2) and one within-subjects factor (3), a significance level of 0.05, a power of 80% and an effect size of 0.75, 19 individuals per group were needed.

## Results

The sociodemographic characteristics of the participants are described in Table 1. At baseline, no significant differences were observed in heart rate (HR) (p = 0.432), oxygen saturation (SpO₂) (p = 0.736), or body mass index (BMI) (p = 0.280) between women with a history of breast cancer and those without. However, women who had been diagnosed with the condition were significantly younger (p < 0.001) and more likely to be childless (p = 0.045).

**Table 1:** Descriptive characteristics of the sample

	BCS (n = 19)^1^	HG (n = 19)^1^	p value^2^	Effect size
Age, years	52.8 ± 5.9	60.3 ± 6.6	< 0.001	1.184
HR, bpm	78.7 ± 10.1	75.5 ± 14.2	0.432	0.258
SpO_2_, %	98 (97, 98)	98 (96.5, 98)	0.736	0.156
BMI, kg/m^2^	22.9 ± 2.6	23.7 ± 2.8	0.280	0.319
Working	17 (89.47%)	11 (57.89%)	0.061	0.299
Children			0.045	0.323
No	7 (36.84%)	1 (5.26%)		
Yes	12 (63.16%)	18 (94.74%)		

^1^Mean ± SD;

^2^Two Sample t-test; Wilcoxon rank sum test; Pearson’s Chi-squared test with simulated p-value (2000 replicates)

Abbreviations: BCS, breast cancer survivors; HG, healthy group; HR, heart rate; bpm, beats per minute SpO2: oxygen saturation; BMI, body mass index. BCS, breast cancer survivors; HG, healthy group

The breast cancer (BC) group consisted of 19 women, all of whom had undergone surgery (100%). In addition, 89.5% (n = 17) received chemotherapy, 84.2% (n = 16) received radiotherapy, and 63.16% (n = 12) presented with lymphedema. Regarding surgical interventions, 89.5% (n = 17) underwent mastectomy, with 52.9% (n = 9) on the right side, 29.4% (n = 5) on the left side, and 17.65% (n = 3) bilaterally.

During follow-up, four participants were lost: one due to worsening of the disease, and three due to relocation.

Table 2 shows the correlations between variables at baseline and at discharge. A significant inverse association was identified between hand grip strength (HGS) and the PIPER Fatigue Scale score among breast cancer survivors at the end of the intervention (p = 0.028). A similar inverse association was also observed between the total PIPER score and the physical health component of the WHOQOL-BREF (p < 0.001).

**Table 2:** Degree of association between variables (Spearman Correlation Coefficient, p-value)

	Breast cancer			Breast cancer	
*HGS*	Yes	No	*Pipper score*	Yes	No
Baseline			Pre		
STS_30	0.20 (0.410)	0.36 (0.135)	WBCV_ PHYS	−0.37 (0.116)	−0.30 (0.208)
QuickDash	0.04 (0.878)	0.00 (1.000)	WBCV_PSYCH	−0.41 (0.082)	−0.36 (0.126)
Pipper score	0.43 (0.063)	−0.03 (0.902)	WBCV_SOC	−0.18 (0.457)	−0.22 (0.360)
			WBCV_ENV	−0.21 (0.396)	−0.41 (0.079)
Post			Post		
STS_30	0.12 (0.621)	0.36 (0.135)	WBCV_PHYS	**-**0.74*	−0.12 (0.621)
QuickDash	0.14 (0.563)	0.12 (0.628)	WBCV_PSYCH	−0.33 (0.166)	−0.06 (0.819)
Pipper score	−0.50 (0.028)*	−0.15 (0.553)	WBCV_SOC	−0.29 (0.237)	−0.13 (0.606)
			WBCV_ENV	−0.36 (0.180)	−0.19 (0.433)

Abreviaton: HGS: Hand Grip Strength; STS_30: 30-Second Sit to Stand Test; QuickDASH: Shortened Disabilities of the Arm, Shoulder and Hand Questionnaire; PIPER score: Piper Fatigue Scale; WBCV_PHYS: WHOQOL-BREF Quality of Life Scale – Physical Dimension; WBCV_PSYCHO: WHOQOL-BREF Quality of Life Scale – Psychological Dimension; WBCV_SOC: WHOQOL-BREF Quality of Life Scale – Social Dimension; WBCV_ENV: WHOQOL-BREF Quality of Life Scale – Environmental Dimension.* p < 0.001.

When analyzing the variables HGS, STS 30, QuickDASH, PIPER score, and the subscales of the WHOQOL-BREF quality of life questionnaire using a multivariate repeated measures model, statistically significant differences were observed for the cancer effect (p = 0.010), the time effect (p < 0.001), and the interaction between them (p = 0.039).

Table 3 presents the results of the univariate repeated measures models. The time factor was significant for all variables, while significant differences between women with and without a history of breast cancer were found only in QuickDASH (p = 0.011) and PIPER score (p < 0.001). Although the interaction was significant in the multivariate model, as previously noted, this appears to be primarily driven by QuickDASH (p = 0.041) and WBCV Physical (p < 0.048). It is likely that, with a larger sample size, the interaction for PIPER score would also reach statistical significance (p = 0.050).

**Table 3:** Results of the univariate repeated measures models from outcome measures

	*Breast Cancer*		*Healthy*		*Group*	Group*Time
	*Baseline*	*Post*	*Baseline*	*Post*		
HGS	21.45 ± 4.70	25.16 ± 4.04	22.82 ± 4.41	26.02 ± 4.82	< 0.001	0.781
STS_30	12.95 ± 2.32	21.21 ± 3.12	13.63 ± 3.48	22.53 ± 5.12	< 0.001	0.605
QuickDash-Sp	21.65 ± 18.44	6.34 ± 7.21	9.21 ± 8.35	1.79 ± 3.08	< 0.001	0.041
Pipper scale,	3.07 ± 2.16	1.35 ± 1.21	0.88 ± 01.26	0.26 ± 0.45	< 0.001	0.050
* Behaviour*	2.19 ± 2.16	0.99 ± 1.17	0.38 ± 0.57	0.12 ± 0.21	0.008	0.067
* Affective*	3.37 ± 2.40	1.78 ± 1.36	0.89 ± 1.35	0.30 ± 0.60	0.004	0.135
* Sensory*	3.57 ± 2.44	1.43 ± 1.53	1.16 ± 1.85	0.39 ± 0.69	< 0.001	0.039
* Cognitive*	3.46 ± 2.08	1.41 ± 1.21	1.24 ± 1.78	0.27 ± 0.53	< 0.001	0.082
WHOQOL-BREF						
* Physical*	62.18 ± 9.11	71.46 ± 9.54	60.61 ± 11.06	78.14 ± 13.55	< 0.001	0.048
* Psicological*	65.41 ± 9.74	72.81 ± 9.66	67.98 ± 9.73	74.34 ± 14.05	< 0.001	0.659
* Social*	59.40 ± 19.12	73.87 ± 14.99	67.98 ± 12.81	76.46 ± 9.37	< 0.001	0.133
* Environment*	70.75 ± 10.70	77.67 ± 10.23	75.66 ± 13.48	80.26 ± 12.77	< 0.001	0.781

HGS: Hand Grip Strength; STS_30: 30-Second Sit to Stand Test; QuickDASH: Shortened Disabilities of the Arm, Shoulder and Hand Questionnaire; PIPER score: Piper Fatigue Scale; WHOQOL-BREF: WHOQOL-BREF: World Health Organization Quality of Life – BREF questionnaire

**Fig. 1:**
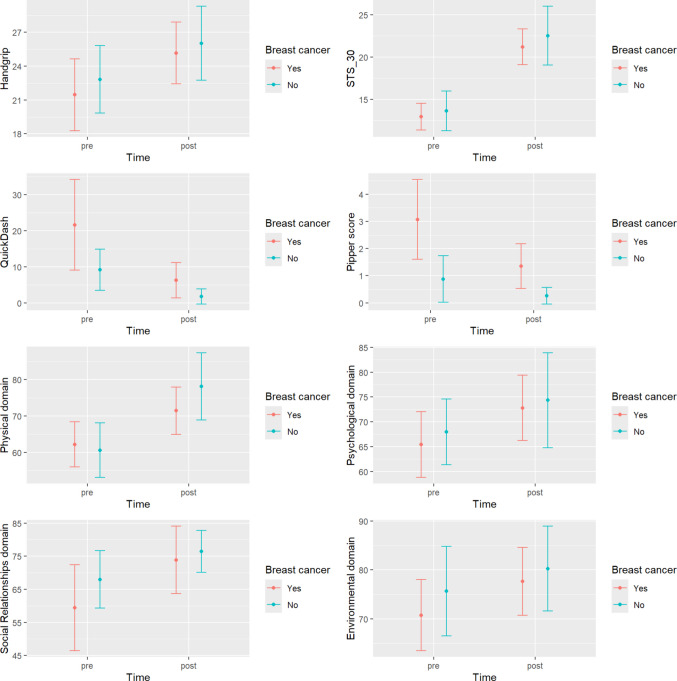
Means and 95% confidence intervals for each univariate repeated measures model

Figure 1 displays the means and 95% confidence intervals for each univariate repeated measures model. The time effect was significant for all variables, indicating a significant mean improvement over time regardless of group (healthy women or breast cancer survivors), except for QuickDASH and PIPER score, where a significant decrease was observed.

However, in the case of QuickDASH, the interaction effect was significant (p = 0.041), indicating that the decrease was not uniform across groups. The reduction in mean score was more pronounced among women who had experienced breast cancer.

For WBCV Physical component, a significant interaction was also detected (p = 0.048), attributable to a more marked mean improvement in the group of women without a history of cancer.

Attendance rate. A high level of participation in the program sessions was observed, with a mean attendance of 20.1 ± 6.7 sessions, exceeding 80% of the total planned sessions (24 in total). In the breast cancer survivors group, the mean attendance was 21.1 ± 4 sessions, corresponding to approximately 85% of the scheduled training sessions. Notably, no participants reported any adverse events—such as discomfort, pain, or injury—related to rowing or strength training activities.

## Discussion

The aim of the present study was to evaluate the effects of a multicomponent intervention program combining falucho rowing with targeted strength and flexibility exercises in both breast cancer survivors and healthy women. The results demonstrate the benefits of this approach in both groups. This study is particularly relevant as it provides insight into the effects of a rowing discipline that has been scarcely explored in the scientific literature. When combined with strength-based cardiovascular physical activity, it offers new opportunities for inclusion in oncology rehabilitation programs [35].

The participants included in this study, both with and without a history of breast cancer, did not show differences in baseline physiological characteristics (HR, SpO₂, BMI), suggesting that both groups were physiologically comparable at a basic level. However, it is noteworthy that women with a history of breast cancer were significantly younger, which may be associated with specific risk factors or earlier diagnosis [36]. In addition, nulliparity was significantly more common among women in this group. This condition may reflect the influence of hormonal and reproductive factors in the development of the disease, which have been identified in the literature as potential protective factors against the onset of this type of cancer [37].

With regard to the variables related to strength and cardiovascular endurance (HGS and STS_30), a significant improvement was observed over time in both groups. This suggests that both variables can improve through a structured physical activity program, regardless of a history of breast cancer.

Having had breast cancer has been associated with an increased risk of cardiovascular events, including death, heart failure, and atrial fibrillation [38]. In this context, strength and endurance training may help reduce the risk of cardiac complications in general, and particularly among breast cancer survivors [39].

These effects have also been previously reported in rowing programs using Dragon Boat vessels. However, from a biomechanical perspective, there are notable differences in rowing technique between the two modalities. In Dragon Boat, participants insert the paddle almost vertically into the water and perform an explosive pushing motion. The hands grip the paddle at the top and mid-shaft to generate propulsion [40].

In contrast, in fixed-seat rowing, the stroke is longer in both time and spatial range. The movement of the upper limbs occurs at shoulder height, involving a pulling motion with both arms executed more symmetrically than the unilateral stroke characteristic of Dragon Boat rowing [41].

On the other hand, the variables disability and fatigue showed significant differences both by cancer diagnosis and in the interaction with time. In the case of disability, as measured by the QuickDASH, the more pronounced decrease observed in women with breast cancer suggests that the program may have been particularly beneficial in improving upper limb function and mobility in this group.

This finding is especially relevant, as functional limitations secondary to breast cancer treatment—including restricted upper limb movement, lymphedema, and fatigue—are common after interventions such as surgery, chemotherapy, or radiotherapy [42]. Physical exercise has been shown to help reduce these complications [43]. Both the gym-based exercises and, notably, the movements involved in rowing contribute to the development of strength and functional mobility, which highlights the value of including this modality in exercise programs for breast cancer survivors [15].

Regarding fatigue, although the interaction effect in this study was close to reaching statistical significance (p = 0.050), it is noteworthy that the program also contributed to improvements in this variable, particularly among women who had experienced breast cancer. Fatigue is a disabling and distressing condition that can affect cancer survivors during the rehabilitation process. This result suggests that the implemented exercise program may have had a positive impact on perceived fatigue, one of the most common and debilitating consequences in this population [44].

Available research demonstrates that exercise is a safe and effective intervention to address cancer-related fatigue. Scott & Posmontier [45] report in their systematic review that supervised group exercise interventions have proven to be highly beneficial for cancer patients in reducing fatigue. Although the impact of group dynamics is not yet fully understood, the social support received and the friendships formed during group-based interventions may play a key role in individuals’ subjective fatigue levels [46]. Given its moderate level of evidence and potential positive effects, the implementation of such approaches would be desirable in oncology rehabilitation programs [47].

With regard to the psychological, social, and environmental dimensions of quality of life, although no significant interaction effects were observed, the positive time effect suggests that engaging in physical activities such as rowing, in a group and outdoor setting, may foster emotional well-being and self-esteem among participants [14, 37], as well as improve adherence to physical activity [48]. Rowing supports the social reintegration of breast cancer survivors by providing an environment of collective motivation and support. Its group-based nature promotes teamwork, as it requires precise synchronization for effective rowing and boat propulsion.

Moreover, participating in the activity alongside women without a cancer diagnosis may be particularly beneficial, promoting a sense of normalcy and reinforcing survivors' self-efficacy [37]. Other group physical activity components, such as social connection, peer support networks, and group cohesion, may have contributed to the improvements observed in the social dimension [12, 49]. The open-sea environment adds a unique environmental element that likely had a positive effect on participants’ perception of well-being and quality of life, underscoring the value of integrating natural settings into interventions [16].

These results should be interpreted with caution due to the methodological limitations of the study. Chief among them is the small sample size, resulting from the specific characteristics of the population and logistical constraints. Nevertheless, this approach aligns with previous research, such as the study by Gavala-González et al. [15], which evaluated the effects of rowing training in breast cancer survivors with a sample of 30 participants [16], and is considered appropriate for exploratory and intervention research in specialized populations.

Second, the duration of the program may have influenced the outcomes, as some participants were still in the process of learning the rowing technique after three months. This could have affected the impact of the program on certain variables, particularly those related to upper limb function and fatigue. It is possible that a longer intervention period and a more refined technique could have led to more pronounced improvements in functional outcomes and quality of life. Nonetheless, the use of the falucho as a training vessel represents an innovative contribution in this field. In the future, adapted rowing training could even be programmed for earlier stages of recovery in breast cancer survivors.

Finally, regarding the data analysis, it is important to note that the use of a robust multivariate approach and bootstrap methods for a moderately sized sample strengthens the validity of the findings. However, future studies with larger samples and longer intervention durations are needed to confirm the observed trends, especially for those variables whose significance values approached the threshold.

## Conclusion

This study provides preliminary evidence supporting the benefits of a multicomponent intervention that combines fixed-seat rowing (falucho) with targeted strength and flexibility exercises in women with and without a history of breast cancer. The intervention was associated with significant improvements in muscular strength, functional mobility, and quality of life. Notably, breast cancer survivors experienced greater reductions in disability and fatigue, highlighting the potential role of such programs in addressing common post-treatment sequelae.

Beyond the physical outcomes, the group-based nature of the intervention, conducted in an outdoor maritime environment, likely contributed to improvements in emotional well-being and social integration. These findings underscore the value of integrating structured, enjoyable, and socially engaging physical activity into oncology rehabilitation.

Future studies with larger sample sizes and longer follow-up periods are warranted to confirm these results and further explore the role of traditional rowing practices as supportive care strategies for cancer survivors.

## Supplementary Information

Below is the link to the electronic supplementary material.

Supplementary file1 (DOCX 1479 KB)

Supplementary file2 (DOCX 29 KB)

## Data Availability

The data that support the findings of this study are available from the corresponding author upon reasonable request.
