# Supplementary material for: Rowing through recovery: Psychophysical outcomes of a combined 12-week rowing and exercise program in breast cancer survivors
Source: Support Care Cancer. 2026 Jan 23;34(2):119. doi: 10.1007/s00520-026-10361-2 (PMC12827310; doi:10.1007/s00520-026-10361-2)
Supplement: Supplementary file 1 — Supplementary file1 (DOCX 1479 KB) [file 520_2026_10361_MOESM1_ESM.docx]

| **LOWER LIMBS** | | | |
| --- | --- | --- | --- |
| **Exercise** | **Exercise description** | **Dosage and load increase** | **Photos** |
| **Gluteus Bands Lying Side** | Patient lies in lateral position, with the elastic band at the distal femur (above the knees). With a 45-degree knee flexion, perform a hip abduction of the upper leg (separate the knees), without separating the feet or making compensatory movements with the body or hips. | W 1-2: 2x10 L_R. R 🡪30’’  W 3-4: 3x10 L_R. R 🡪30’’  W 5-6: 3x10 M_R. R 🡪30’’  W 7-8: 4x10 M_R. R 🡪30’’  W 9-12: 4x10 H_R. R 🡪30’’ | **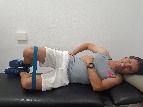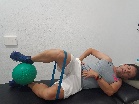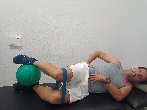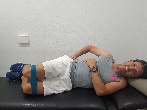** |
| **Gluteus Bands – Bipodal Glute Bridge** | Patient in supine position, with the elastic band at the distal femur (above the knees). Starting from a 90-degree knee flexion, perform the glute bridge, supporting both legs separated at the same height as the shoulders. Keeping the hip stability in line with the body, perform a hip abduction. | W 1-2: 2x10 L_R. R 🡪30’’  W 3-4: 3x10 L_R. R 🡪30’’  W 5-6: 3x10 M_R. R 🡪30’’  W 7-8: 4x10 M_R. R 🡪30’’  W 9-12: 4x10 H_R. R 🡪30’’ | **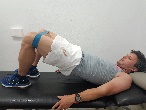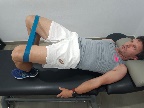** |
| **Gluteus Bands – Monopodal Glute Bridge** | Patient in supine position, with the elastic band at the distal femur (above the knees). Starting from a 90-degree knee flexion, perform the glute bridge, supporting both legs separated at the same height as the shoulders. Keeping the hip stability in line with the body, perform a hip abduction, extending one leg, maintaining the initial abduction and without the hip falling. Alternating one leg and another. | W 1-2: 2x10 L_R. R 🡪30’’  W 3-4: 3x10 L_R. R 🡪30’’  W 5-6: 3x10 M_R. R 🡪30’’  W 7-8: 4x10 M_R. R 🡪30’’  W 9-12: 4x10 H_R. R 🡪30’’ | **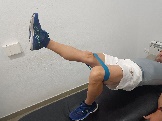** |
| **Gluteus Bands 5-Meter Forward and Backward Displacement** | Patient standing, with the elastic band at the distal femur (above the knees). Starting from a 30-degree knee flexion and 45-degree trunk flexion with a straight back, perform displacements forward 5 meters and then back, without allowing the feet to come together, maintaining tension in the elastic band. Avoid compensations with the body that should not sway. | W 1-2: 2x30'' displacing 5 m forward and backward L_R, Ankle bands, R 30''  W 3-4: 3x30'' displacing 5 m forward and backward L_R, Ankle bands, R 30''  W 5-6: 3x30'' displacing 5 m forward and backward M_R, Ankle bands, R 30''  W 7-8: 4x30'' displacing 5 m forward and backward M_R, Ankle bands, R 30''  W 9-10: 4x30'' displacing 5 m forward and backward H_R, Ankle bands, R 30''  W 11-12: 5x30'' displacing 5 m forward and backward H_R, Ankle bands, R 30'' | 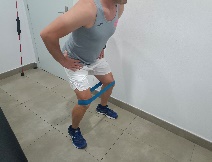 |
| **Gluteus Bands 5-Meter Lateral Displacement** | Patient standing, with the elastic band at the distal femur (above the knees). Starting from a 30-degree knee flexion and 45-degree trunk flexion with a straight back, perform lateral displacements of 5 meters, without allowing the feet to come together, maintaining tension in the elastic band. Avoid compensations with the body that should not sway. | W 1-2: 2x30'' displacing 5 m laterally, L_R, Ankle bands, R 30''  W 3-4: 3x30'' displacing 5 m laterally, L_R, Ankle bands, R 30''  W 5-6: 3x30'' displacing 5 m laterally, M_R, Ankle bands, R 30''  W 7-8: 4x30'' displacing 5 m laterally, M_R, Ankle bands, R 30''  W 9-10: 4x30'' displacing 5 m laterally, H_R, Ankle bands, R 30''  W 11-12: 5x30'' displacing 5 m laterally, H­_R, Ankle bands, R 30'' | 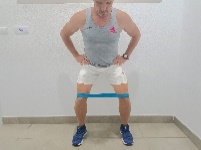 |
| **Isometric Hamstring with Fitball - Gluteus Bridge** | Patient in supine position, starting from a 90-degree knee flexion, performs the glute bridge supporting the feet on the fitball, maintaining hip stability in line with the body and maintaining the position. | W 1-2: 2x15’’ isométrico. R 🡪30’’  W 3-4: 2x20’’ isométrico. R 🡪30’’  W 5-6: 2x30’’ isométrico. R 🡪30’’  W 7-8: 3x30’’ isométrico R 🡪30’’  W 9-12: 3x40’’ isométrico. R 🡪30’’ | 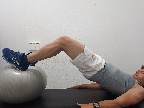 |
| **Dynamic Hamstring with Fitball - Gluteus Bridge** | Patient in supine position, starting from a 90-degree knee flexion, performs the glute bridge supporting the feet on the fitball, maintaining hip stability in line with the body, extends and bends knees. | W 1-2: 2x5. R 🡪30’’  W 3-4: 2x8. R 🡪30’’  W 5-6: 3x8. R 🡪30’’  W 7-8: 3x10. R 🡪30’’  W 9-12: 3x12 . R 🡪30’’ | 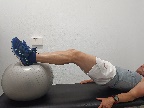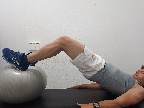 |
| **Squats with elastic bands** | Patient standing, stepping on the elastic bands with the feet and holding them with the hands, the patient will perform a knee flexion up to 90 degrees. | W 1-2: 2x10 L_R. R 🡪30’’  W 3-4: 3x10 L_R. R 🡪30’’  W 5-6: 3x10 M_R. R 🡪30’’  W 7-8: 4x10 M_R. R 🡪30’’  W 9-12: 4x10 H_R. R 🡪30’’ | 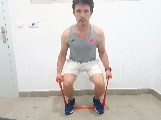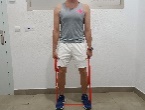 |
| **Dymanic straight lunge** | Patient standing with hands on the hip for better stability, will perform a lunge forward, flexing the knee between 45 and 90 degrees. Will alternate one leg and the other performing the repetitions according to the phase we are in. | W 1-2: 2x6 with each leg, R 30''  W 3-4: 2x8 with each leg, R 30''  W 5-6: 3x8 with each leg, R 30''  W 7-8: 3x10 with each leg, R 30''  W 9-12: 3x12 with each leg, R 30'' | 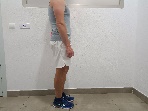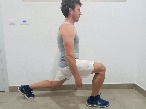 |
| **Dynamic lateral lunge** | Patient standing with hands interlaced in front of the body, will perform a lateral lunge flexing the knee between 45 and 90 degrees. Will alternate one leg and the other performing the repetitions according to the phase we are in. | W 1-2: 2x6 with each leg, R 30''  W 3-4: 2x8 with each leg, R 30''  W 5-6: 3x8 with each leg, R 30''  W 7-8: 3x10 with each leg, R 30''  W 9-12: 3x12 with each leg, R 30'' | 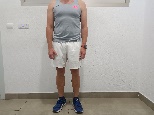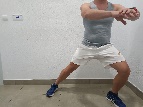 |

**W, weeks; R, resistance; L_R, light resistance; M_R, medium resistance; H_R, high resistance; R, recovery**

| **TRUNK** | | | |
| --- | --- | --- | --- |
| **Exercise** | **Exercise description** | **Dosage and load increase** | **Photos** |
| **Abdominals - activation rectus abdominis** | Patient in supine position on a mat, with legs flexed at 90 degrees and feet against the floor. Place hands laterally to the neck (without forcing or pulling on it) and stick the chin to your chest rolling your body until the scapulae come off the floor. While performing the rolling movement, you must exhale progressively by puckering the lips. | W 1-2: 2x15. R 🡪30’’  W 3-4: 3x15. R 🡪30’’  W 5-6: 3x20. R 🡪30’’  W 7-8: 4x20. R 🡪30’’  W 9-12: 4x25. R 🡪30’’ | 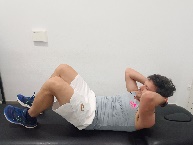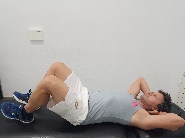 |
| **Prone Crunch - activation transverse abdominis** | Patient in prone position (lying face down), supporting the body on the forearms and knees on the floor (later we will only support the feet). Perform a contraction of the abdomen aligning the body and hips while performing a scapular approach. | W 1-2: 2x20'' Knees supported, R 30''  W 3-4: 2x30'' Knees supported, R 30''  W 5-6: 2x30'' Feet supported, R 30''  W 7-8: 3x30'' Feet supported, R 30''  W 9-12: 3x40'' Feet supported, R 1' | **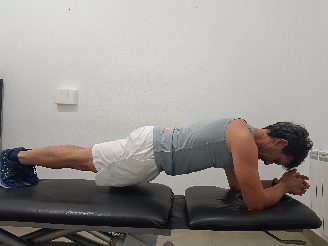** |
| **Lateral Crunch - activation abdominal obliques** | Patient in lateral position, supporting the body on the forearm of the floor side and the knee of the same side on the floor (later we will only support the feet). Perform a contraction of the abdomen aligning the body and hips laterally. | W 1-2: 2x20'' Knees supported, R 30''  W 3-4: 2x30'' Knees supported, R 30''  W 5-6: 2x30'' Feet supported, R 30''  W 7-8: 3x30'' Feet supported, R 30''  W 9-12: 3x40'' Feet supported, R 1' | 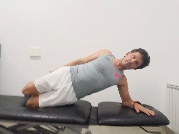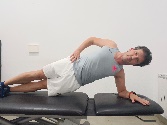 |
| **Superman or Lumbar Extensions** | Lying face down, we will perform an extension of the trunk (lifting the arms) and simultaneous legs. For better gluteal activation, we will put the elastic bands between the ankles. | W 1-2: 2x5. R 🡪30’’  W 3-4: 2x8. R 🡪30’’  W 5-6: 3x8. R 🡪30’’  W 7-8: 3x10. R 🡪30’’  W 9-12: 3x12. R 🡪30’’ | 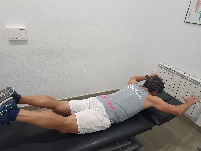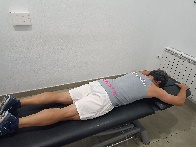 |

**W, weeks; R, resistance; L_R, light resistance; M_R, medium resistance; H_R, high resistance; R, recovery**

| **UPPER LIMBS** | | | |
| --- | --- | --- | --- |
| **Exercise** | **Exercise description** | **Dosage and load increase** | **Photos** |
| **Isometric Elastic Bands - 90º Elbows in Front** | Patient in sitting or standing position, with the elastic bands in the proximal part of the forearms or wrists (according to evolution). Starting from a position of 90-degree elbow flexion attached to the body, maintain the position of the arms with tension in the bands and simultaneously, perform a scapular retraction. Maintain the position isometrically the time according to evolution. | W 1-2: 2x20’’ L_R on wrists. R 🡪30’’  W 3-4: 3x20’’ L_R on wrists. R 🡪30’’  W 5-6: 3x20’’ M_R on wrists. R 🡪30’’  W 7-8: 4x20’’ M_R on wrists. R 🡪30’’  W 9-10: 4x20’’ H_R on wrists. R 🡪30’’  W 11-12: 5x20’’ H_R on wrists. R 🡪30’’ | **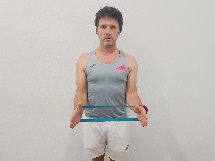** |
| **Isometric Elastic Bands - Straight Elbows in Front** | Patient in sitting or standing position, with the elastic bands in the proximal part of the forearms or wrists (according to evolution). Starting from a position of elbow extension and 90-degree shoulder flexion, maintain the position of the arms with tension in the bands and simultaneously, perform a scapular retraction. Maintain the position isometrically the time according to evolution. | W 1-2: 2x20’’ L_R on wrists. R 🡪30’’  W 3-4: 3x20’’ L_R on wrists. R 🡪30’’  W 5-6: 3x20’’ M_R on wrists. R 🡪30’’  W 7-8: 4x20’’ M_R on wrists. R 🡪30’’  W 9-10: 4x20’’ H_R on wrists. R 🡪30’’  W 11-12: 5x20’’ H_R on wrists. R 🡪30’’ | **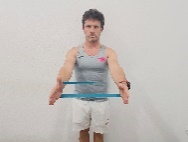** |
| **Isometric Elastic Bands - 90º Elbows Behind** | Patient in sitting or standing position, with the elastic bands in the proximal part of the forearms or wrists (according to evolution). Starting from a position of shoulder extension, 90-degree elbow flexion, maintain the position of the arms with tension in the bands. Maintain the position isometrically the time according to evolution. | W 1-2: 2x20’’ L_R on wrists. R 🡪30’’  W 3-4: 3x20’’ L_R on wrists. R 🡪30’’  W 5-6: 3x20’’ M_R on wrists. R 🡪30’’  W 7-8: 4x20’’ M_R on wrists. R 🡪30’’  W 9-10: 4x20’’ H_R on wrists. R 🡪30’’  W 11-12: 5x20’’ H_R on wrists. R 🡪30’’ | 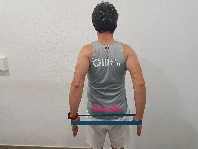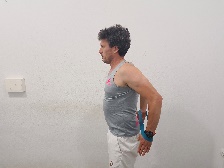 |
| **Isometric Elastic Bands - Straight Elbows Behind** | Patient in sitting or standing position, with the elastic bands in the proximal part of the forearms or wrists (according to evolution). Starting from a position of shoulder and elbow extension, maintain the position of the arms with tension in the bands. Maintain the position isometrically the time according to evolution. | W 1-2: 2x20’’ L_R on wrists. R 🡪30’’  W 3-4: 3x20’’ L_R on wrists. R 🡪30’’  W 5-6: 3x20’’ M_R on wrists. R 🡪30’’  W 7-8: 4x20’’ M_R on wrists. R 🡪30’’  W 9-10: 4x20’’ H_R on wrists. R 🡪30’’  W 11-12: 5x20’’ H_R on wrists. R 🡪30’’ | 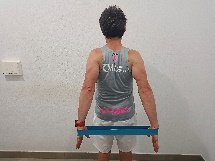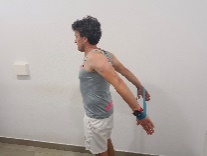 |
| **Dynamic Elastic Band Shoulder Movements in Front** | Patient in sitting or standing position, with the elastic bands in the proximal part of the forearms or wrists (according to evolution). Starting from a position of elbow extension and 90-degree shoulder flexion, maintaining tension in the elastic bands, perform shoulder flexion movements, circles in one direction and another. | W 1-2: 2x10 L_R. R 🡪30’’  W 3-4: 3x10 L_R. R 🡪30’’  W 5-6: 3x10 M_R. R 🡪30’’  W 7-8: 4x10 M_R. R 🡪30’’  W 9-10: 4x10 H_R. R 🡪30’’  W 11-12: 5x10 H_R. R 🡪30’’ | **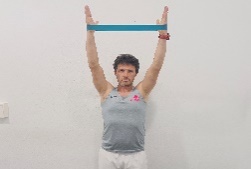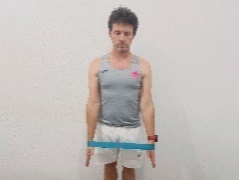** |
| **Dynamic Elastic Band Shoulder Movements Behind** | Patient in sitting or standing position, with the elastic bands in the proximal part of the forearms or wrists (according to evolution). Starting from a position of elbow and shoulder extension, maintaining tension in the elastic bands, perform shoulder extension movements, circles in one direction and another. | W 1-2: 2x10 L_R. R 🡪30’’  W 3-4: 3x10 L_R. R 🡪30’’  W 5-6: 3x10 M_R. R 🡪30’’  W 7-8: 4x10 M_R. R 🡪30’’  W 9-10: 4x10 H_R. R 🡪30’’  W 11-12: 5x10 H_R. R 🡪30’’ | 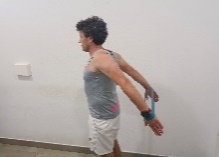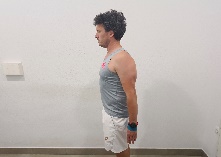 |
| **External Rotation (90º-0º)** | Patient standing with slight knee and hip flexion to the side of the band anchor. With the elbow attached to the body and a 90-degree elbow flexion, performs a concentric contraction of external rotators using an elastic band. | W 1-2: 2x10 L_R. R 🡪30’’  W 3-4: 3x10 L_R. R 🡪30’’  W 5-6: 3x10 M_R. R 🡪30’’  W 7-8: 4x10 M_R. R 🡪30’’  W 9-10: 4x10 H_R. R 🡪30’’  W 11-12: 5x10 H_R. R 🡪30’’ | 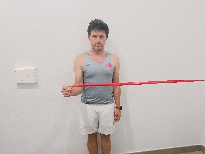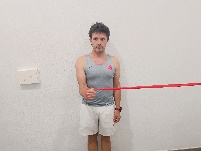 |
| **Internal Rotation (90º-0º)** | Patient standing with slight knee and hip flexion to the side of the band anchor. With the elbow attached to the body and a 90-degree elbow flexion, performs a concentric contraction of internal rotators using an elastic band. | W 1-2: 2x10 L_R. R 🡪30’’  W 3-4: 3x10 L_R. R 🡪30’’  W 5-6: 3x10 M_R. R 🡪30’’  W 7-8: 4x10 M_R. R 🡪30’’  W 9-10: 4x10 H_R. R 🡪30’’  W 11-12: 5x10 H_R. R 🡪30’’ | 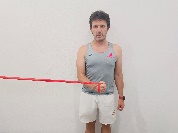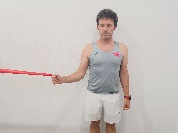 |
| **Eccentric external rotators** | Patient standing with slight knee and hip flexion, facing the band anchor, with the shoulder in 90-degree abduction and elbow flexed 90º. An external rotation is performed in a rapid concentric contraction to subsequently perform an eccentric contraction (slow and controlled return). | W 1-2: 2x10 L_R. R 🡪30’’  W 3-4: 3x10 L_R. R 🡪30’’  W 5-6: 3x10 M_R. R 🡪30’’  W 7-8: 4x10 M_R. R 🡪30’’  W 9-10: 4x10 H_R. R 🡪30’’  W 11-12: 5x10 H_R. R 🡪30’’ | 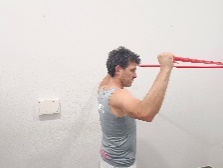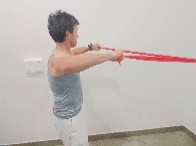 |
| **Low row with elastic bands** | Patient standing with a slight trunk flexion with extended arms and scapular protraction. The final position of the exercise will be with elbow flexion and scapular retraction. | W 1-2: 2x10 L_R. R 🡪30’’  W 3-4: 3x10 L_R. R 🡪30’’  W 5-6: 3x10 M_R. R 🡪30’’  W 7-8: 4x10 M_R. R 🡪30’’  W 9-10: 4x10 H_R. R 🡪30’’  W 11-12: 5x10 H_R. R 🡪30’’ | 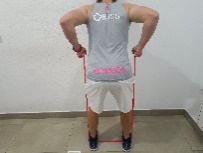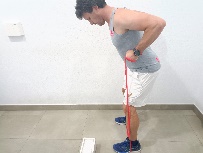 |
| **Robbery exercise.** | Patient standing facing the band anchor, with elbows flexed.  Performs a bilateral external rotation of rapid contraction and return to the slow starting position. | W 1-2: 2x10 L_R. R 🡪30’’  W 3-4: 3x10 L_R. R 🡪30’’  W 5-6: 3x10 M_R. R 🡪30’’  W 7-8: 4x10 M_R. R 🡪30’’  W 9-10: 4x10 H_R. R 🡪30’’  W 11-12: 5x10 H_R. R 🡪30’’ | 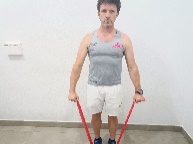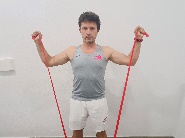 |
| **Scaption** | Patient standing with slight knee and hip flexion. Holding an elastic band with the hands, while stepping on it with the feet, performs an abduction up to 90º with thumbs up. Along with a horizontal flexion of 30º. | W 1-2: 2x10 L_R. R 🡪30’’  W 3-4: 3x10 L_R. R 🡪30’’  W 5-6: 3x10 M_R. R 🡪30’’  W 7-8: 4x10 M_R. R 🡪30’’  W 9-10: 4x10 H_R. R 🡪30’’  W 11-12: 5x10 H_R. R 🡪30’’ | 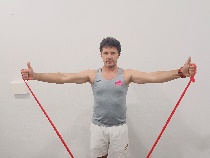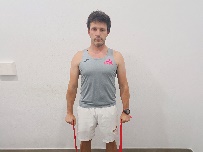 |
| **Chest with Elastic Bands** | Patient standing with arms in 90-degree abduction and maximum extension (elbows extended) and legs slightly flexed. The exercise is performed by holding the band with the hands and bringing it forward until the straight arms are joined in front of the body. | W 1-2: 2x10 L_R. R 🡪30’’  W 3-4: 3x10 L_R. R 🡪30’’  W 5-6: 3x10 M_R. R 🡪30’’  W 7-8: 4x10 M_R. R 🡪30’’  W 9-10: 4x10 H_R. R 🡪30’’  W 11-12: 5x10 H_R. R 🡪30’’ | 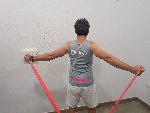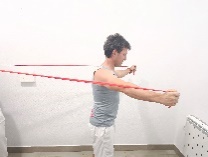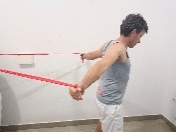 |
| **Push up plus.** | Patient performs a ‘push up’ adding a protraction/retraction scapular. The patient starts in a scapular protraction followed by a retraction. A push up is performed maintaining the scapular retraction. | W 1-2: 2x6 Knees supported. R 🡪30’’  W 3-4: 2x8 Knees supported. R 🡪30’’  W 5-6: 2x10. Knees supported. R 🡪30’’  W 7-8: 2x10 Feet supported. R 🡪30’’  W 9-12: 3x10 Feet supported. R 🡪1’ | 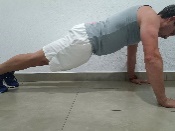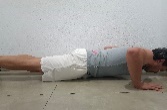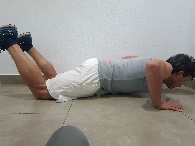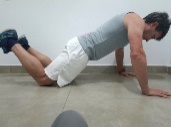 |

**W, weeks; R, resistance; L_R, light resistance; M_R, medium resistance; H_R, high resistance; R, recovery**
