# Supplementary material for: Rowing through recovery: Psychophysical outcomes of a combined 12-week rowing and exercise program in breast cancer survivors
Source: Support Care Cancer. 2026 Jan 23;34(2):119. doi: 10.1007/s00520-026-10361-2 (PMC12827310; doi:10.1007/s00520-026-10361-2)
Supplement: Supplementary file 2 — Supplementary file2 (DOCX 29 KB) [file 520_2026_10361_MOESM2_ESM.docx]

Appendix 2. Exercise Reporting.

| **CERT item** |  |
| --- | --- |
| **1. Equipment** | Minimal: Elastic bands and fit balls. |
| **2. Instructor qualifications** | Physiotherapist (More than 15 years expertise) |
| **3. Individual or group** | Two groups (19 Brest Cancer and 19 Healthy women). |
| **4. Supervised or not** | Supervised by physiotherapist in the gym and rowing (12 weeks) |
| **5. Adherence** | Group work was done to increase adherence. |
| **6. Motivation** | They were given a dossier with images and explanation of the exercises for each participant and the sessions were supervised by an expert physiotherapist. |
| **7. Progression** | 12 weeks, with a gradual progression in volume and intensity. This progression was achieved by progressively increasing the number of repetitions and the resistance of the elastic bands. |
| **8. Exercise description** | See Appendix 1 (type, reps, rest, hold). |
| **9. Home program** | The program was made in the club. |
| **10. Setting** | The exercises were performed in the club: both groups participated in fixed-seat rowing sessions of 60 minutes each twice a week and 12-week program of therapeutic exercises (flexibility and muscle strength) – Appendix 1. |
| **11. Generic/individually tailored** | Generic. Specific corrections were made for each subject during the rowing and therapeutic exercise sessions. |
| **12. Starting level rule** | The intervention included strength, endurance and mobility exercises, targeting the trunk, lower and upper limbs. |
